# Supplementary material for: Cellular and synaptic phenotypes lead to disrupted information processing in Fmr1-KO mouse layer 4 barrel cortex
Source: Nat Commun. 2019 Oct 23;10:4814. doi: 10.1038/s41467-019-12736-y (PMC6811545; doi:10.1038/s41467-019-12736-y)
Supplement: Supplementary file 1 — Supplementary Information [file 41467_2019_12736_MOESM1_ESM.pdf]

Supplementary material to accompany Domanski *et al.* 2019:

Cellular and Synaptic Phenotypes Lead to Disrupted Information Processing in Fmr1-KO Mouse Layer 4 Barrel Cortex

Contents:

- *7 Supplementary figures*
- *1 Supplementary table*
- *Supplementary Discussion and further references*

Supplemental Figures

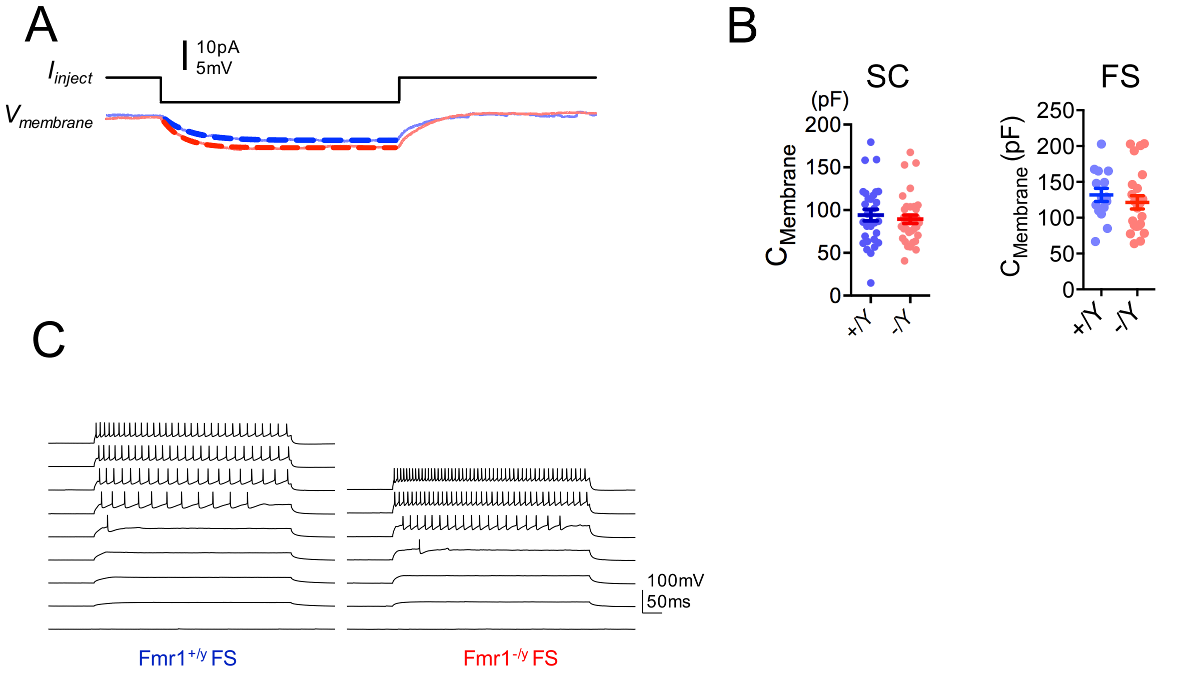

Supplemental Figure 1

Passive properties of P10-11 WT and *Fmr1-KO* layer 4 SC and FS neurons.

A). Example data showing procedure for calculating passive properties of neurons from current clamp data. Neurons were maintained at -60mV, close to rest. Small hyperpolarising current steps were applied to produce negative membrane potential deflections. Thick dotted lines indicate single exponential fits used to derive membrane time constants.

B). Whole-cell membrane capacitance of layer 4 SCs and FS neurons. No significant differences were observed between genotypes for either cell ($p>0.05$, t-test. Data shows mean±SEM. Symbols indicate individual neurons).

C). FS interneuron intermediate trace examples (P10 animals, Δ100pA current steps from -60mV holding potential to 2x rheobase current).

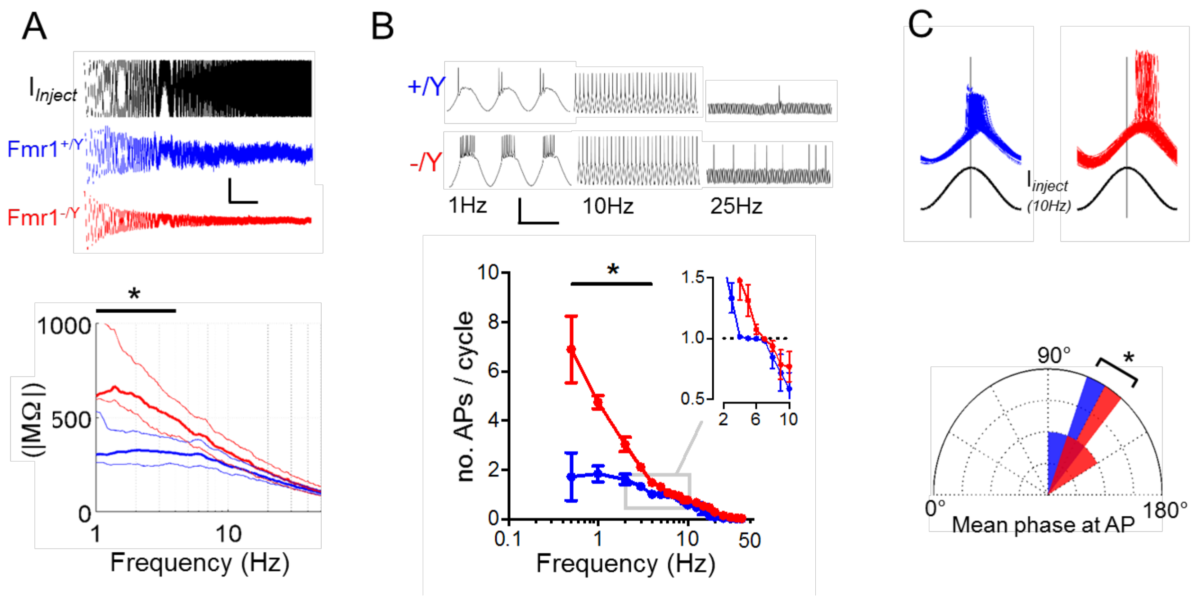

Supplemental Figure 2

Altered resonance properties in *Fmr1-KO* SCs cause increased excitability and a phase shift in response to low frequency depolarizations

A). Top: Subthreshold frequency responses of layer 4 SCs. Example membrane potential deflections (blue/red) in response to injection of constant amplitude sinusoidal 'ZAP' function (black) with a 25s linear frequency ramp from 0.5Hz to 50Hz (scale: 2.5s/10mV). Bottom: Impedance profiles (mean±95% C.I.s.) for layer 4 SCs. Impedance values were significantly different for frequencies below 4Hz (p=0.02, Mann-Whitney (n: 6 WT, 6 *Fmr1-KO*).

B). Frequency locking of action potential firing to oscillatory current injections by layer 4 excitatory neurons. Top: Example AP firing of WT (above) and *Fmr1-KO* (below) neurons in response to 5s of 80pA (peak to peak) current injection for constant frequencies of 1, 10 and 25Hz (Scale: 50mV/1s). Bottom: Number of APs fired per cycle for current injections between 0.5Hz and 50Hz (mean±SEM). Firing rates differed significantly for input frequencies between 0.5-4Hz (p<0.05, Mann-Whitney (n: 5 WT, 8 *Fmr1-KO*). Inset: Expanded view of frequency range over which WT neurons lock to one AP per cycle (4-7Hz in WT neurons vs. 7Hz in *Fmr1-KO* neurons, P>0.05, one-sample t–test).

C). Phase preference of AP firing for layer 4 SCs. Top: Membrane potential (WT and *Fmr1-KO* in blue and red, respectively, 200 cycles overlaid) is shown aligned to phase of injected current (black). Bottom: Histograms showing mean instantaneous phase (Hilbert transform) of injected current at AP peak. Phase preference was significantly different between genotypes at 10Hz (WT: 112±4.4°, *Fmr1-KO*: 129±5.3° p=0.039, t-test, N=5 animals for each

genotype) but not 7Hz (Not shown (WT: 95.5±2.9°, *Fmr1-KO*: 92.0±5.2° p=0.59). Here, peak of oscillation is at 90° for each genotype**.**

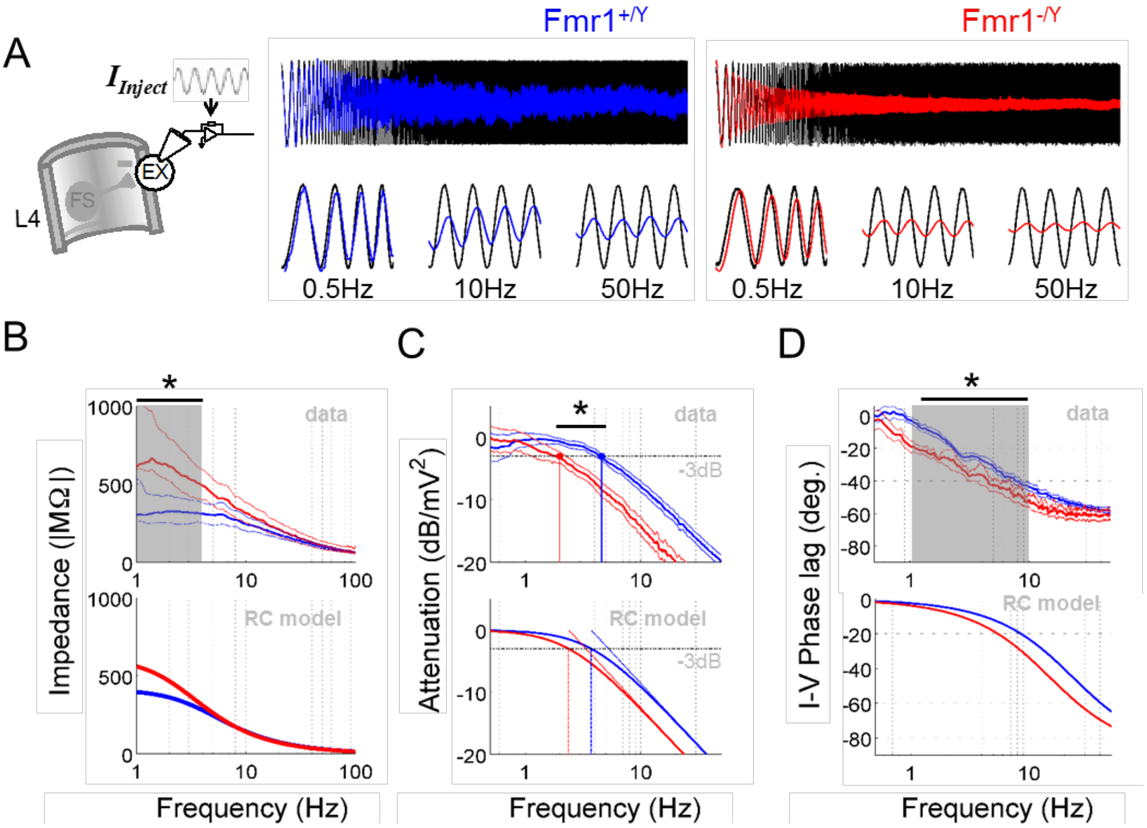

Supplemental Figure 3

Intrinsic electrical pathophysiology alters frequency processing by P10-11 *Fmr1-KO* layer 4 SCs.

A). Subthreshold frequency responses of layer 4 excitatory neurons. Example membrane potential deflections (blue/red) in response to injection of constant amplitude sinusoidal 'ZAP' function (black) with a 20s linear frequency ramp from 0.5Hz to 50Hz.

B). Impedance profiles (mean±95% C.I.s.) for layer 4 excitatory neurons (top) and theoretical values for a passive low-pass RC filter assuming genotype mean parametersd for $R_{membrane}$ and $C_{membrane}$ (bottom). Impedance values were significantly different for frequencies below 4Hz (p=0.02, Mann-Whitney (n: 6 *Fmr1$^{+/Y}$*, 6 *Fmr1$^{-/Y}$*), Grey shaded region).

C). Bode (gain) profiles showing $V_{membrane}$ oscillatory power attenuation from DC for data and theoretical curves shown in **B).** -3dB cut-off frequencies were significantly lower by ~3Hz in the *Fmr1-KO* data (shown by asterisk, vertical lines in top plot, statistics as **B).**). Filter roll-off

slope (angled lines in lower, theoretical plot) were unaltered and consistent with a one-pole low pass filter (20dB/decade attenuation).

D). Current-voltage phase shifts determined through Fourier analysis were significantly greater in *Fmr1-KO* neurons compared to those from wild-types by an additional 10 degrees as averaged between 1 and 10Hz (statistics as **B).**).

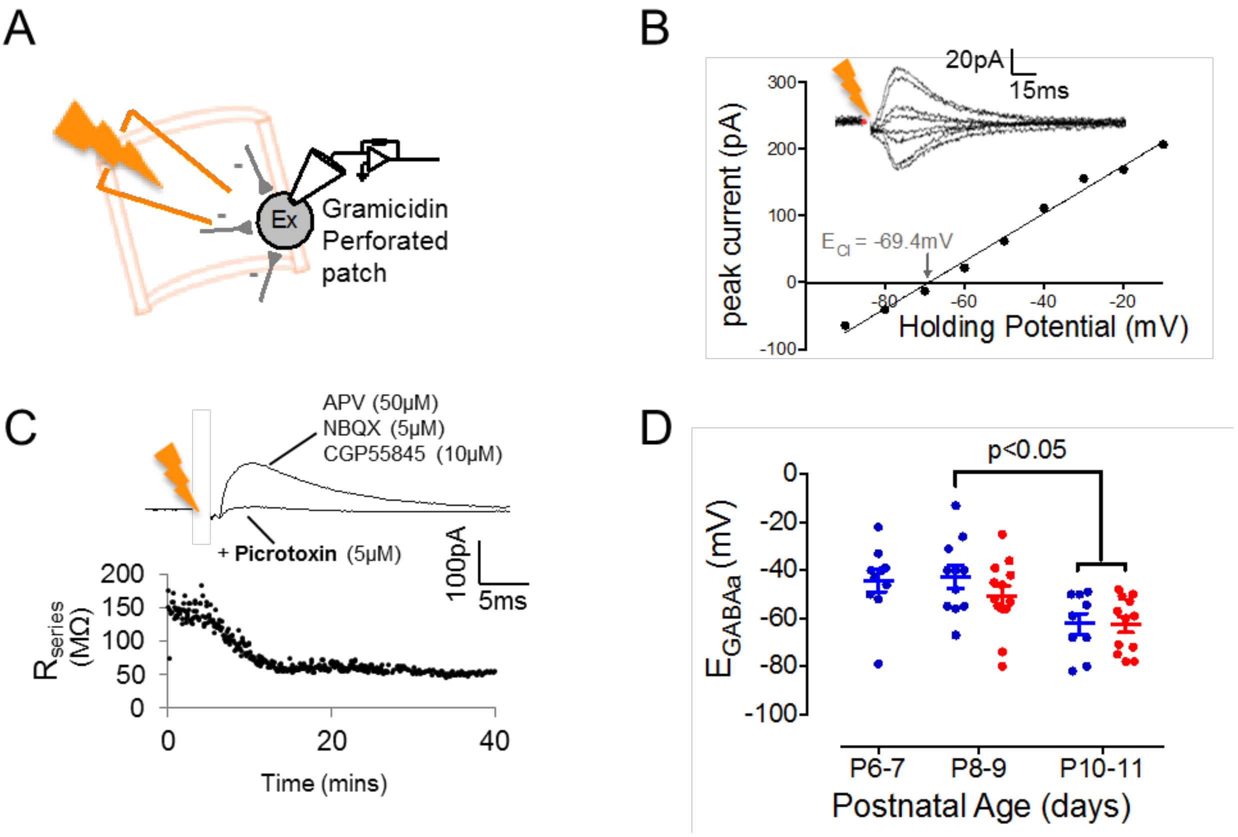

Supplemental Figure 4

Normal [Cl$^-$]$_{internal}$ development in *Fmr1-KO* SCs

A). Schematic showing isolation of GABA$_A$ reversal potential (E$_{cl}$) by gramicidin perforated patch voltage clamp recording and bulk stimulation of local synaptic inputs.

B). Current-voltage relationship of evoked GABAergic currents in an example recording (P10, *Fmr1*$^{-/Y}$) showing linear fit to peak current amplitudes at holding potential between -90 and -10mV.

C). Top: Example evoked GABAergic currents (P11 *Fmr1*$^{+/Y}$, V$_{holding}$=-40mV) showing blockade by picrotoxin. Bottom: Stability of example perforated patch recording showing series resistance during 30 minute recording period following initial patch stabilization.

D). Day-by-day developmental progression of E$_{cl}$ between P6 and P11 for *Fmr1*$^{+/Y}$ (blue) and *Fmr1*$^{-/Y}$ (red) neurons. E$_{cl}$ for neurons of both genotypes was significantly more hyperpolarized at P10-11 compared to *Fmr1*$^{+/Y}$ neurons at P7-8. No other pair-wise comparisons were significantly different, including P8-9 *Fmr1*$^{+/Y}$ vs. P8-9 *Fmr1*$^{-/Y}$, or P10-11 *Fmr1*$^{+/Y}$ vs. P10-11 *Fmr1*$^{-/Y}$ (two-way ANOVA with Tukey's test for multiple comparisons, Individual points are neurons (max. 2 per animal), bars are mean±SEM).

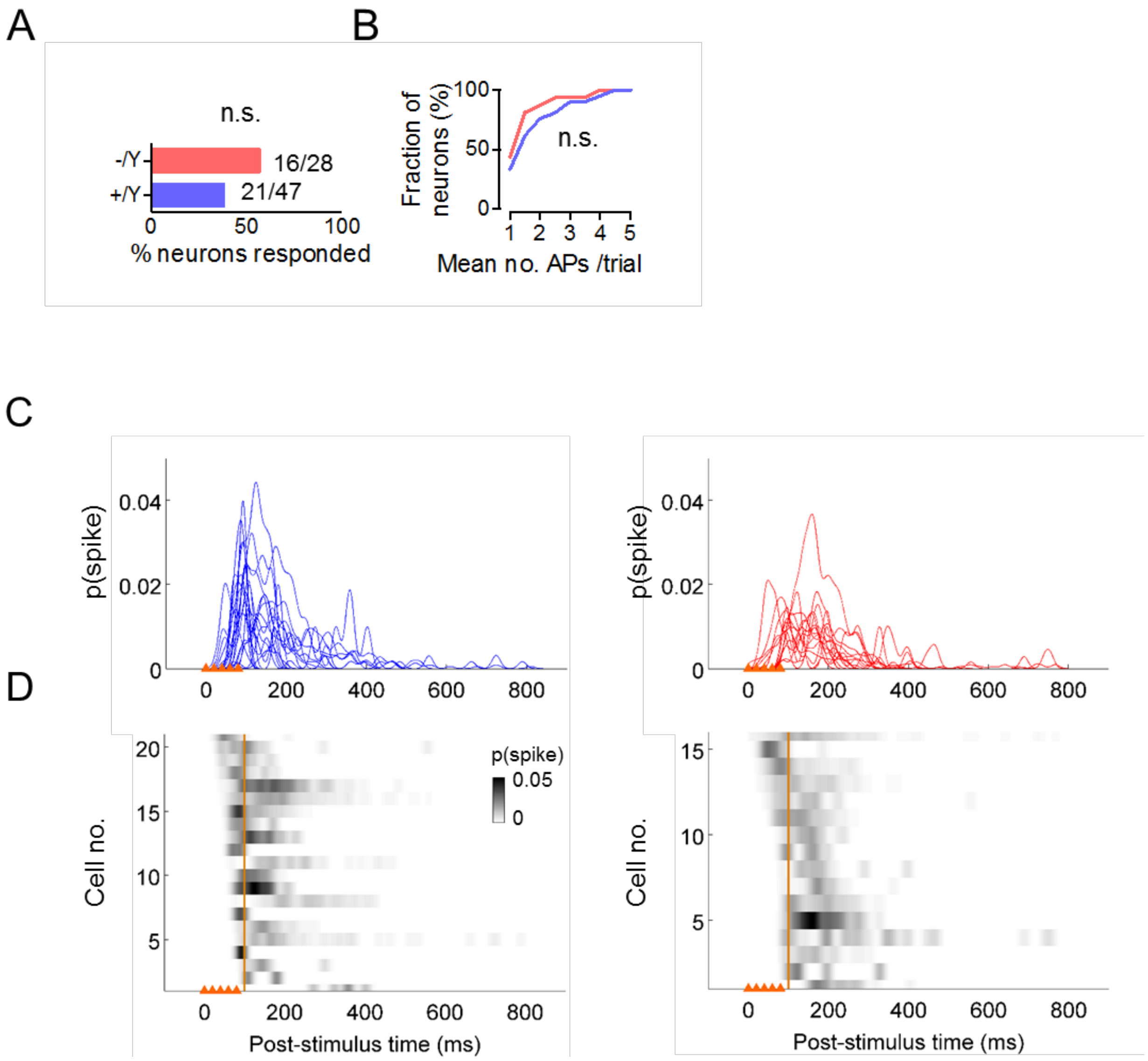

Supplemental Figure 5

Participation of layer 4 SCs in TC-evoked network activity

Quantification of spiking activity from cell-attached recordings. Number of neurons analysed in this dataset: *Fmr1*$^{+/Y}$= 21, *Fmr1*$^{+/Y}$= 16, maximum of 3 from each animal.

A). Fraction of layer 4 SCs participating in network response was not significantly different between genotypes (χ^2 test, p>0.05).

B). Average numbers of spikes fired per trial were not significantly different between genotype (K-S test, p>0.05).

C). Mean spike probability density functions (5ms kernel standard deviation) for 21 *Fmr1*$^{+/Y}$ and 16 *Fmr1*$^{-/Y}$ neurons (maximum of 3 per animal).

D). Expanded view of spike probability density for neurons shown in **C)**, ordered from top to bottom by time of first spike onset.

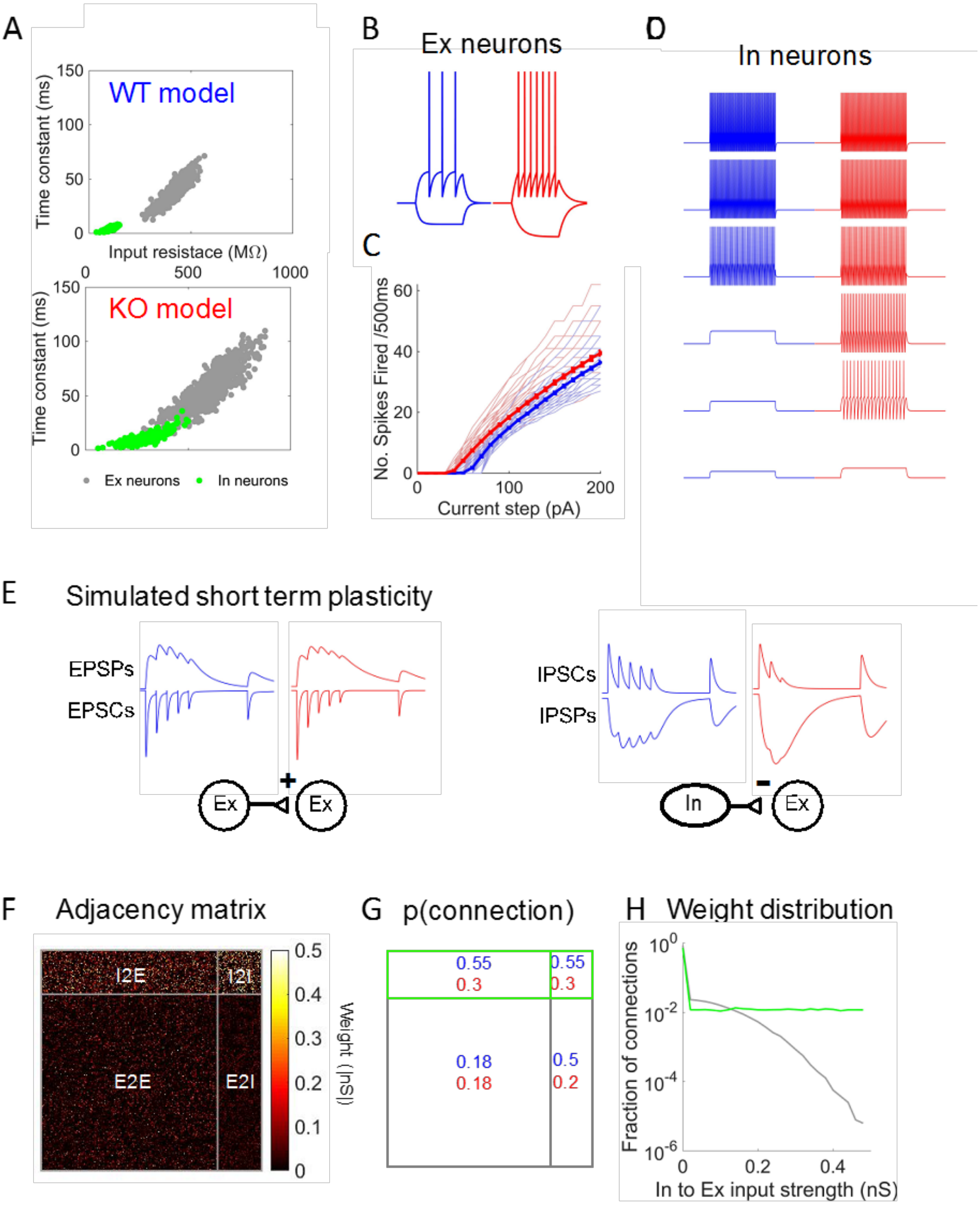
A
WT model
KO model
Time constant (ms)
Input resistace (MΩ)
Ex neurons
In neurons
B
Ex neurons
C
No. Spikes Fired /500ms
Current step (pA)
D
In neurons
E
Simulated short term plasticity
EPSPs
EPSCs
IPSCs
IPSPs
Ex
Ex
In
Ex
F
Adjacency matrix
I2E
I2I
E2E
E2I
Weight (nS)
G
p(connection)
0.55
0.3
0.55
0.3
0.18
0.18
0.5
0.2
H
Weight distribution
Fraction of connections
In to Ex input strength (nS)

Supplemental Figure S6

Generation of a randomly connected model of a simplified layer 4 barrel

A). Distributions of model neuron intrinsic properties were drawn from multivariate Gaussian distributions describing the experimentally-derived values for Input resistance and membrane time constants.

B). Simulated voltage responses to identical square current pulses for Ex neurons. Note the greater voltage deflection and elevated spike count for the *Fmr1-KO* model.

C). Simulated F-I curves for 20 model neurons of each genotype. Note lower rheobase in *Fmr1-KO* model.

D). Simulated firing response for example FS model neurons of each genotype to increasing amplitude current steps.

E). Example synapse-specific simulated short-term plasticity between model connected neuron pairs during 50Hz stimulation and a remote test pulse.

F). Example weight matrix describing model network connectivity for one random seed. “I2I” reflects Inhibitory to inhibitory synapses, “E2I” reflects excitatory to inhibitory connectivity, etc.

G). Connection probability between different classes of neurons. Blue (red) numbers indicate connected fraction for each synapse type in the WT (*Fmr1-KO*) model.

H). Example synaptic weight distributions for excitatory (grey, lognormal distribution) and inhibitory (green) inputs to Excitatory cells.

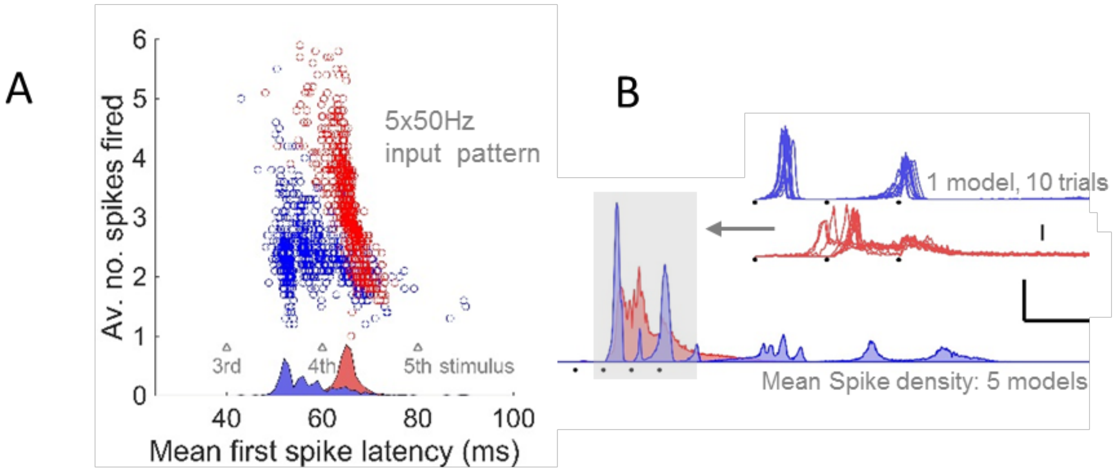

Supplemental Figure 7

Spike time and rate changes in population response of *Fmr1-KO* layer 4 model network to simulated TC input

A). Delayed first spike onset times in the *Fmr1-KO* model. Ex neuron firing patterns: Example first spike statistics and spike counts from WT (blue) and *Fm1-KO* (red) simulations. (10 repeated runs of one model random seed each). Each circle is one Ex neuron. Histograms on abscissa summarise first spike latency. Note later response onset in KO model. Note that the first two stimuli (t=0,20s) are not shown.

B). Grand mean Ex. spike density histograms for WT and *Fm1-KO* models (5 random seeds, 10 repeats each). Note lower population synchrony in *Fm1-KO* model. Inset expands grey shaded region: individual trial repeats for one WT and KO random seed. Note delayed and variable onset population response in KO model. Scale: 50ms, 10% Ex neuron synchrony.

Supplemental table 1 – Layer 4 network model parameters

Intrinsic and synaptic parameters

	Units	WT Ex	WT In	KO Ex	KO In
No. neurons	N/A	800	150	800	150
$R_{membrane}$	MΩ*	**410(±50)**	**100(±20)**	**600(±100)**	**300(±80)**
$C_{membrane}$	pF*	90(±10)±10	40(±5)±10	90(±10)±15	40(±5)±10
V_{rest}	mV	-65	-65	-65	-65
$V_{threshold}$	mV	-45	-45	-45	-45
V_{peak}	mV	35	20	35	20
V_{reset}	mV	-65	-85	-65	-85
$E_{[NMDA/AMPA]}$	mV	0	0	0	0
$E_{[Cl-]}$	mV	-71	-71	-71	-71
NMDA Gate "Alpha"	mV^{-1}	0.062	0.062	0.062	0.062
NMDA Gate "Beta"	mM	3.57	3.57	3.57	3.57
$Mg^{2+}_{[out]}$	mM	1.2	1.2	1.2	1.2
Synaptic delay	ms	0.5	0.5	0.5	0.5
τ_{rise} AMPA	ms	0.6	0.1	0.6	0.1
τ_{decay} AMPA	ms	0.3	1	0.3	1
τ_{rise} NMDA	ms	3	N/A	3	N/A
τ_{decay} NMDA	ms	75	N/A	75	N/A
τ_{rise} GABA	ms	**0.7**	**8**	**1**	**1**
τ_{decay} GABA	ms	**0.7**	**8**	**12**	**12**

* Values shown represent mean (±[shared variance]) ±[private variance] where shared variance between $R_{membrane}$ and $C_{membrane}$ underlies membrane time constant.

Numbers in **bold** indicate parameters different between WT and KO models.

Synaptic Connectivity
Connection probability / Strength

	Units	WT	KO
$p_{(Ex \rightarrow Ex)}$ / $G_{max[AMPA]}$	% / nS	18/0.0001	18/0.0001
$p_{(Ex \rightarrow Ex)}$ / $G_{max[NMDA]}$	% / nS	18/0.0001	18/0.0001
$p_{(Ex \rightarrow In)}$ / $G_{max[AMPA]}$	% / nS	**50/0.0001**	**20/0.00015**
$p_{(In \rightarrow Ex)}$ / $G_{max[GABA]}$	% / nS	**55**/0.0002	**33**/0.0002
$p_{(In \rightarrow In)}$ / $G_{Max[GABA]}$	% / nS	**50**/0.0005	**33**/0.0005
$p_{(TC \rightarrow Ex)}$ / $G_{max[AMPA]}$	% / nS	80/0.005	80/0.005
$p_{(TC \rightarrow Ex)}$ / $G_{max[NMDA]}$	% / nS	80/0.005	80/0.005
$p_{(TC \rightarrow In)}$ / $G_{max[AMPA]}$	% / nS	80/0.025	80/0.025

Short term plasticity
% depression per stim / $\tau_{recovery}$

	Units	WT	KO
Ex → Ex	% / ms^{-1}	60 / **2000**	60 / **5000**
Ex → In	% / ms^{-1}	**45** / 500	**20** / 500
In → Ex	% / ms^{-1}	60 / **500**	60 / **3000**
In → In	% / ms^{-1}	60 / **500**	60 / **3000**
TC → Ex	% / ms^{-1}	**70** / 500	**50** / 500
TC → In	% / ms^{-1}	70 / **2000**	70 / **8000**

Supplemental discussion

Relationship of this work to previous literature on Feed Forward information processing in the *Fmr1-KO* brain

Notably, Wahlstrom-Helgren and Klyachko (2015)[1] have previously demonstrated aberrant FFI in the cortico-hippocampal feedforward circuit of P21 *Fmr1-KO* mice. The FFI alterations were synaptic pathway-specific and were attributed to altered presynaptic $GABA_B$ function. As in the present study, the authors reported a steady-state evoked EPSP broadening and an activity-dependent reduction in spike precision by the postsynaptic cell that required synaptic interactions in a two-pathway integration paradigm. We now demonstrate a role for altered postsynaptic intrinsic excitability in determining an aberrant functional response of the postsynaptic cell following presynaptic stimulation and extend the study to evaluate the ramification of altered FFI activity at the network level in somatosensory cortex. We also demonstrate distorted excitatory thalamocortical short-term plasticity contributes to the altered summation properties of layer 4 SCs. Short-term depressing synapses provide gain control and temporal filtering mechanisms that favour information transmission at lower frequencies [2,3]. We provide evidence for a shift in temporal and rate input discrimination in *Fmr1-KO* layer 4 that we attribute, in part, to stronger STP of depressing synapses. Notably, although these mechanisms could be linked by a common dependence on intracellular calcium signalling, Deng and Klyachko (2013)[4] report a diminished information transmission capacity at facilitating synapses of hippocampal CA3 neurons that most dramatically alters processing of high frequency information. Wahlstrom-Helgren & Klyachko (2016)[5] further describe a detailed mechanism for the activity-dependent broadening of EPSPs and IPSPs in the Schaffer collateral hippocampal pathway. They report that precise timing and dynamic strength changes regulate activity-dependent EPSP broadening, similar to what we report here for single-pathway summation at the thalamocortical synapse in somatosensory cortex. Finally, using experimentally verified simulations they demonstrate that effects on spike probability and precision are controlled independently by short-term plasticity of excitation and inhibition, respectively. Therefore both our findings and those of Wahlstrom-Helgren & Klyachko (2016)[5] suggest a link between steady-state E/I balance and activity-dependent EPSP broadening.

Potential biophysical mechanisms underlying cell-type divergent regulation of firing rate in response to injected current

Interestingly, we found that while both SC and FS cells in the *Fmr1-KO* recordings displayed elevated intrinsic excitability, they displayed divergent firing rate alterations to injected current stimuli. One plausible scenario underlying this bidirectional modulation of firing rate could be a parallel cell-type specific alteration to voltage-dependent conductances in the SC and FS

cells, ultimately manifesting as a broader AP in both cell types: Compared to WT SC neurons, AP repolarization speed was slower in the *Fmr1-KO* SCs. In contrast, *Fmr1-KO* FS neurons displayed reductions in both spike depolarization and repolarization speed. These extra effects in FS neurons could offset the elevated input resistance and impair their ability to rapidly fire trains of APs.

Using modelling to identify causal vs. compensatory network-level contributions to network dysfunction

Our modelling approaches have the potential to reduce cost, time and experimental animal usage. Supportive of this aim, as a companion to this paper we provide user-friendly simulation code with full documentation and usage tutorials to encourage non-specialists to experiment with the function of the circuits studied herein, as well as adapt them for their own use (see Methods for hosting repository links).

To further address the question of causal vs. compensatory physiological disruptions, three main approaches will be necessary, firstly through physiological investigations spanning development time-points (this study and references [6,7]) to evaluate the developmental evolution of features of physiological dysfunction, possibly in combination with chronic sensory manipulation (e.g. whisker deprivation) or carefully timed transient rescue interventions. This approach can be augmented by (secondly) analytical mathematical methods and (thirdly) numerical simulations. The derivation of exact analytical expressions governing network activity is notably challenging in the face of the complex, non-linear circuit dynamics [8,9] but reduced circuit or parametric statistical models (e.g. reference [10]) offer powerful insight into which parameter(s) are dominant and which the network is robust to upon manipulation [10,11]. Numerical simulation offers a complementary approach to studying network homeostasis and development [12,13]. These crucially provide predictions for the stochastic evolution of network structure and dynamics under different combinations of interventions, thereby comparing simulated network developmental trajectories from a common starting point. We introduce here a machine learning approach to classifying stimulus identity from multi-neuronal firing patterns. This could be extended to probe the evolving information capacity of model networks at different stages of *in silico* development. One promising approach is to compare a (model) circuit's observed information capacity to that of a theoretically optimal model, in which a trade-off has been met for factors such as neural response correlation and response redundancy [14]. The derivation of such an optimal circuit model could be hampered however by the animals' adoption of alternative behavioural strategies to extract relevant information from sensory input, as has been reported in Autistic individuals [15,16].

Future experiments combining experimental manipulations of activity levels with model predictions will be able to elucidate which cellular parameters remain malleable and hence what pharmacological interventions may be more effective in restoring circuit function. These predictions can subsequently undergo rigorous testing by physiological experimentation. Such an approach would also be a valuable tool for examining the convergence of pathophysiology in disease mechanisms affecting circuit function across a range of genetic models.

Limitations and potential extensions of models used in this study

Although our *in silico* models provide both mechanistic insight into and strong predictions about the quality of L4 circuit dysfunction in *Fmr1-KO* animals, it is important to recognise their limitations. Firstly, our two models lack 3D dendritic structure or detailed information about dendritic integration and thus only represent a first-order approximation of point summation at the soma. As such, potential changes to compartmentalised summation of thalamocortical input affecting active dendritic integration [17] or functional clustering of synaptic input could have been inadvertently overlooked. Moreover, we focused on genotype means of parameter sets grouped for simplicity by rational biophysical mechanisms. Consequently, we may have missed subtle antagonistic effects between collapsed parameters. For example, the changes in short-term plasticity we report in the experimental data are most likely attributable to presynaptic alterations that could potentially represent distinct changes in quantal release probability and/or failure rate that we capture in a single term in our phenomenological model of short-term depression. By providing the modelling code and analysis tools alongside this manuscript we encourage re-use of the simulations to both gain further intuitive insight into circuit function and genotype-dependent change and support novel analyses of similar datasets. One intriguing extension of our "rescue scenario" modelling approach could be to use dynamic clamp [18] to play in rescued *Fmr1-KO* currents into patch recordings from wild-type neurons (and vice versa). This could offer both validation of our findings and an opportunity to disentangle direct compensation vs. causative effects. For example, by both elevating the tone of Feed-forward inhibitory input to a wild-type SC and artificially reducing its leak conductance, one could examine whether an *Fmr1-KO* SC –like response emerged at a single-cell level, thereby removing changes to short-term plasticity from the potential causative mechanisms and isolating potential slower network-dependent compensations. Modelling in this setting should not be used as a stand-alone tool. The above example represents one of several possible opportunities to integrate modelling as a predictive tool into a physiological experimental pipeline. Further detailed modelling efforts could include distributed inputs to the SC dendritic arbour and would ideally be accompanied by large-scale detailed Ca^{2+} imaging of synaptic input to the SC neurons and systematic sampling of single-synaptic input strength [19].

References

1. Wahlstrom-Helgren, S. & Klyachko, V. A. GABA $_B$ receptor-mediated feed-forward circuit dysfunction in the mouse model of fragile X syndrome. *J. Physiol.* **593,** 5009–5024 (2015).

2. Tsodyks, M., Pawelzik, K. & Markram, H. Neural Networks with Dynamic Synapses. *Neural Comput.* **10,** 821–835 (1998).

3. Tsodyks, M. V & Markram, H. The neural code between neocortical pyramidal neurons depends on neurotransmitter release probability. *Proc. Natl. Acad. Sci. U. S. A.* **94,** 719–23 (1997).

4. Deng, P.-Y. *et al.* FMRP Regulates Neurotransmitter Release and Synaptic Information Transmission by Modulating Action Potential Duration via BK Channels. *Neuron* **77,** 696–711 (2013).

5. Wahlstrom-Helgren, S. & Klyachko, V. A. Dynamic balance of excitation and inhibition rapidly modulates spike probability and precision in feed-forward hippocampal circuits. *J. Neurophysiol.* **116,** 2564–2575 (2016).

6. Gibson, J. R., Bartley, A. F. A., Hays, S. A. & Huber, K. M. Imbalance of neocortical excitation and inhibition and altered UP states reflect network hyperexcitability in the mouse model of fragile X syndrome. *J. Neurophysiol.* **100,** 2615–2626 (2008).

7. Harlow, E. *et al.* Critical Period Plasticity Is Disrupted in the Barrel Cortex of Fmr1 Knockout Mice. *Neuron* **65,** 385–398 (2010).

8. Transtrum, M. K., MacHta, B. B. & Sethna, J. P. Why are nonlinear fits to data so challenging? *Phys. Rev. Lett.* **104,** 2–5 (2010).

9. O'Leary, T., Sutton, A. C. & Marder, E. Computational models in the age of large datasets. *Curr. Opin. Neurobiol.* **32,** 87–94 (2015).

10. Panas, D. *et al.* Sloppiness in Spontaneously Active Neuronal Networks. *J. Neurosci.* **35,** 8480–8492 (2015).

11. Machta, B. B., Chachra, R., Transtrum, M. K. & Sethna, J. P. Parameter Space Compression Underlies Emergent Theories and Predictive Models. *Science (80-.).* **4878,** 3192–3195 (2013).

12. O'Leary, T., Rossum, M. C. W. Van, Wyllie, D. J. a, O'Leary, T. & van Rossum, M. C. W. Homeostasis of intrinsic excitability in hippocampal neurons: dynamics and mechanism of the response to chronic depolarization. *J. Physiol.* **1,** 157–170 (2009).

13. Litwin-Kumar, A. & Doiron, B. Formation and maintenance of neuronal assemblies through synaptic plasticity. *Nat. Commun.* **5,** 1–12 (2014).

14. Tkacik, G., Prentice, J. S., Balasubramanian, V. & Schneidman, E. Optimal population coding by noisy spiking neurons. *Proc. Natl. Acad. Sci.* **107,** 14419–14424 (2010).

15. Happé, F., Frith, U. & B, P. T. R. S. The beautiful otherness of the autistic mind. *Philos. Trans. R. Soc. B* 1345–1350 (2009). doi:10.1098/rstb.2009.0009

16. Livingston, L. A. & Happé, F. Conceptualising compensation in neurodevelopmental disorders: Reflections from autism spectrum disorder. *Neurosci. Biobehav. Rev.* **80,** 729–742 (2017).

17. Lavzin, M., Rapoport, S., Polsky, A., Garion, L. & Schiller, J. Nonlinear dendritic processing determines angular tuning of barrel cortex neurons in vivo. *Nature* 5–9 (2012). doi:10.1038/nature11451

18. Sharp, A. A., O'Neil, M. B., Abbott, L. F. & Marder, E. Dynamic clamp: computer-generated conductances in real neurons. *J. Neurophysiol.* **69,** 992–995 (1993).

19. Ashby, M. C. & Isaac, J. T. R. Maturation of a recurrent excitatory neocortical circuit by experience-dependent unsilencing of newly formed dendritic spines. *Neuron* **70,** 510–21 (2011).
